# Perspectives of education sector stakeholders on a teacher training module to reduce HIV/AIDS stigma in Western Kenya

**DOI:** 10.1186/s12889-021-11331-5

**Published:** 2021-06-30

**Authors:** Ashley Chory, Winstone Nyandiko, Whitney Beigon, Josephine Aluoch, Celestine Ashimosi, Dennis Munyoro, Michael Scanlon, Edith Apondi, Rachel Vreeman

**Affiliations:** 1 Department of Global Health and Health Systems Design, Arnhold Institute for Global Health, Icahn School of Medicine at Mount Sinai, 1216 Fifth Avenue, Fifth Floor, Room 556, New York, NY USA; 2 Academic Model Providing Access to Healthcare (AMPATH), Eldoret, Kenya; 3 Department of Child Health and Paediatrics, School of Medicine, College of Health Sciences, Moi University, Eldoret, Kenya; 4 Indiana University School of Medicine, Indianapolis Indiana, USA; 5 Moi Teaching and Referral Hospital, Eldoret, Kenya

**Keywords:** HIV, Stigma, Teacher training, Adolescents, Kenya

## Abstract

**Background:**

For adolescents living with HIV (ALWH), school may be the most important but understudied social sphere related to HIV stigma. Teachers are role models in the classroom and within the community, and their attitudes and behavior towards people living with HIV may have critical psychosocial and treatment ramifications. Altering teachers’ knowledge, attitudes and beliefs (K/A/B) about HIV could reduce the stigmatizing content within their teaching, classrooms and school, improving the environment for ALWH.

**Methods:**

We developed a one-day teacher training module to enrich teacher K/A/B that included lecture presentations, HIV films and educational animation, structured instructions for teacher role play scenarios, and a question-and-answer session facilitated by a trained ALWH peer educator. We also conducted key informant interviews with education sector subject matter experts (SMEs), including education officers, county commissioners and head teachers to review and provide feedback on the teacher training module.

**Results:**

We assembled an adolescent community advisory board and recruited 50 SMEs to review the training module and provide feedback. All SME participants stressed the importance and need for interventions to reduce stigma in the classroom, highlighting their own experiences observing stigmatizing behaviors in the community. The participants perceived the training as culturally relevant and easy to understand and had minor suggestions for improvement, including using image-based resources and brighter colors for ease of reading. All participants thought that the training should be expanded outside of the schools, as all people in a community have a role in the reduction of HIV stigma, and offered suggestions for other settings for implementation.

**Conclusion:**

Data from interviews with education sector stakeholders demonstrate that our process for developing a culturally appropriate multi-media intervention to reduce HIV stigma in the schools was feasible.

**Supplementary Information:**

The online version contains supplementary material available at 10.1186/s12889-021-11331-5.

## Background

HIV- and AIDS-related stigma (HIV stigma) shapes every aspect of care management for adolescents living with HIV (ALWH), including medication adherence, decisions about HIV disclosure, mental health and how they grow to take responsibility for their own care [1]. HIV stigma is associated with worse adherence, delayed HIV disclosure, and worse mental health, all of which hinder retention in care and viral suppression [1–6]. For ALWH, school may be the most important but understudied social sphere related to HIV stigma. In the school setting, youth may experience HIV stigma related to their own status or to the HIV status of a family member [1], and may affect not only treatment outcomes but also academic performance, student-teacher relationships, and peer networks. ALWH report experiencing HIV-related taunting, gossiping or bullying by peers at school [7–9] that may lead to problems in attendance or accessing peer support networks [10, 11]. In Kenya, both adolescents and their caregivers report that HIV stigma in schools negatively affects their retention in care, adherence to medications, mental health, and beliefs about themselves [1, 12]. ALWH report experiencing HIV stigma from their teachers directly, as well as indirectly from components of the current HIV curriculum that are stigmatizing or contain false information [6, 12]. Not only do school-based experiences of HIV stigma directly impact ALWH, but stigmatized content and negative teacher attitudes shape the beliefs of their peers regarding HIV and its treatment [13]. Adolescents spend most of their waking hours in school, yet there are few studies of intervention strategies to reduce HIV stigma in this setting.

Teachers are role models in the classroom, school and within the community, and their attitudes and behavior toward people living with HIV (PLWH) may have critical psychosocial and treatment ramifications. Stigmatizing attitudes not only negatively impact ALWH, but may also influence the behaviors and attitudes of un-infected youth. Despite HIV stigma experiences in the classroom, there is very little data on whether stigmatizing attitudes could be altered through teacher-targeted interventions. A recent systematic review [14] of interventions targeting teachers in an effort to reduce classroom HIV stigma described a significant gap in the literature, identifying only two relevant studies [15, 16].

A HIV stigma reduction intervention targeting teachers in South Africa [15] compared an interactive CD-ROM based training discussing common HIV related topics and a two-day workshop that included a combination of lectures and role-play scenarios. Both training groups received information on common topics including HIV transmission, risk factors for infection, and actions teachers can take when facing HIV related challenges in the classroom; a significant reduction in overall HIV stigma was observed, with no difference between the study groups [15]. Norr and colleagues implemented a peer facilitated HIV stigma reduction intervention targeting primary school teachers in training in Malawi [16]. A sub-set of teacher-participants were engaged in a six-week training program that provided education on common HIV topics and then asked to facilitate a full day training for their peers. Despite significant reductions in HIV stigmatizing beliefs overall, the majority (59%) of teacher participants still agreed with stigmatizing opinions like ‘it’s a disgrace if you find out that someone in your family has HIV/AIDS.’ [16] These studies demonstrated a significant reduction in HIV stigma among its participants, suggesting the feasibility and potentially effective nature of the approach.

Enriching and altering teachers’ knowledge, attitudes and beliefs (K/A/B) about HIV could reduce the stigmatizing content within their teaching, classrooms and schools, improving the environment for ALWH. We sought to develop a locally adapted, multi-media teacher-training module designed to decrease negative HIV-related K/A/B and HIV stigma among primary and secondary school teachers in western Kenya. Here, we describe the rigorous process of development and qualitative evaluation of the teacher training curriculum, which involved education sector subject matter expert (SME) engagement. This work is intended to provide insight into culturally appropriate intervention development, as well as a qualitative inquiry into the issues with addressing HIV stigma in school settings in Kenya.

## Methods

### Development of the teacher training module

We sought to develop a teacher-training curriculum or module that could be administered during a one-day training review. Introducing media and interactions that both increase knowledge about HIV infection and engage empathy and emotional connection around the *stories* of life with HIV, may alter teacher knowledge and attitudes about HIV, which may subsequently impact stigmatizing behavior overall and in their classrooms. We based development of these modelson a framework for “empathic technologies” to augment human interaction, targeting an increase in teachers’ “cognitive empathy” – in this case, their understanding of how people living with HIV might feel – through specific, personal disclosures of the challenges of living with HIV, both through filmed “disclosures” of characters’ inner lives and the sharing of lived experiences by youth living with HIV [17]. In addition, we wanted to follow aspects of social learning theory for the content of this teacher training module (‘Teach HADITHI module’), seeking to leverage existing, evidence-based content for decreasing HIV stigma. Social learning posits learning is an active, social process. The social environment, cognitive processes, and behaviors are all mutually interdependent (“reciprocal determinism”), so behaviors are influenced by the social environment and by an individual’s own cognitive processes [18, 19]. Behaviors, once realized, can spread to others through observation of the behavior and its consequences (“vicarious reinforcement”). In this case, attempting to further influence the complex social interactions mediated by the teacher in their classrooms around how people living with HIV are or should be treated.

We searched for existing materials on HIV stigma or stigma reduction interventions through systematic review of the literature [20], and also conducted a critical review of existing materials, books, and curricula used to teach about HIV in schools. In addition, our study team had previously developed various counseling and educational materials focused around children living with HIV for use in the clinical setting in western Kenya, including short films about children living with HIV and facing stigma, educational pamphlets, and an animation explaining HIV physiology and treatment [21]. We wanted to evaluate whether any of these materials could also be incorporated into the Teach HADITHI Module.

Following our systematic review of the literature; our critical review of available local, national, and global HIV-related curriculum and teacher training materials; and evaluation of our locally developed multimedia resources, we assembled a proposed Teach HADITHI Module that included lecture presentations, HIV films and the educational animation, structured instructions for teacher role play scenarios, and a question-and-answer session facilitated by a trained ALWH who serves as a peer educator on the study team. (Table 1) The components of this training module are described in more detail below.

**Table 1:** Teacher Training Module Components

Module Section	Content	Activity Type	Conceptual Framework
Slide presentations	Mechanism for facilitating the training and introducing major themes to be discussed.	Visual	
HIV Animation	Short animated films depicting the biological processes associated with HIV infection and treatment.	Visual	Cognitive Factors: Knowledge
HIV Booklet	Paper booklet featuring frequently asked questions and responses about HIV and common myths and misconceptions.	Take-away materials for independent reading	Cognitive Factors: Knowledge
HIV Films	Culturally adapted short-films depicting the stories of ALWH in school and with their families in Kenya. (www.hiv-films.org)	Visual and Group Interactive	Cognitive Empathy & Social Learning Theory: Parasocial interaction
Case Scenarios	Teacher participants to be presented with HIV specific case scenarios to read and discuss.	Group interactive	Social Learning Theory: Modeling, Efficacy
Teacher role play	Breakout sessions in which teachers would be asked to navigate common scenarios depicting HIV disclosure when working with ALWH. Training participants would play the role of the teacher and a peer navigator living with HIV on the study team would play the adolescent.	Group interactive	Social Learning Theory: Modeling, Efficacy
Experience sharing	Adolescent peer navigator living with HIV on the study team presents real life experiences in the school setting.	Personal sharing by ALWH	Cognitive Empathy & Social Learning Theory: Parasocial interaction

#### HIV animation video

The HIV animation video was developed through qualitative inquiry and consultation with multiple pediatric care providers in western Kenya. Explanatory metaphors and storyboard illustrations were created in Adobe Illustrator CS6. Adolescents ages 12–18 and enrolled in care at AMPATH participated in focus group discussions (FGDs) to assess the acceptability and effectiveness of the animations. The animations were subsequently used in post-disclosure counseling sessions with children living with HIV to explain the immunologic and physiologic complexities of HIV at an elementary level [22]. The HIV animation video would be played in the beginning of the training to provide teachers a basic level of HIV understanding prior to continuing with more interactive and discussion-based sections. The animation video aimed to provide accurate knowledge about HIV infection and transmission, to provide the foundation for the knowledge that would then be applied through social learning theory and understood through the empathetic lens.

#### HADITHI booklet

The HADITHI booklet is a paper resource that was previously developed by the study team through a review of literature and HIV related educational materials. The booklet includes a list of frequently asked questions and evidence-based responses presented in lay terminology. Among the list of questions included in the booklet are: ‘How did I get the HIV virus?,’ ‘How long do I need to take the medicines?,’ and ‘Can this disease be cured?’ The HADITHI booklet also includes a section on myths and misconceptions, presented as commonly heard statements about HIV and evidence-based clarifications to the myth, followed by a breakdown of basic HIV related facts. Among the list of myths and misconceptions are: ‘HIV can be spread during contact with saliva, such as through kissing or the sharing of utensils,’ ‘HIV infections can be cured by having sex with a virgin,’ and ‘Getting HIV/AIDS is a death sentence.’ The HADITHI booklet will be given to teacher-participants to review and take back to their communities. The HADITHI booklet aims to dispel dangerous myths related to HIV in an effort to ease concerns based in misinformation and potentially influence how one interacts with PLWH, again providing the appropriate, factual cognitive content.

#### HIV stigma films

The HADITHI films [23] were previously created by the research team through a collaborative, community participatory process. We conducted 6 FGDs regarding HIV stigma with 40 adolescents (mean age 13 years) and 53 caregivers of HIV-infected children from 3 HIV clinics in western Kenya, as well as key informant interviews with community participatory board members (e.g. caregivers, HIV-infected children, shop keepers, pastors and teachers.) The most significant theme among youth was HIV stigma experienced in the school setting, following by HIV stigma enacted via public ridicule and shunning in social settings, as well as extreme social isolation. Our multidisciplinary, multinational team (which included a filmmaker, anthropologist, sociologist, child development specialists, and pediatricians, both American and Kenyan) used the qualitative analyses to create 4 culturally sensitive, context-focused, narrative films that portray HIV-infected adolescents experiencing HIV stigma at home, clinic, school, and church. The narrative medium intends to not only convey information, but engage an empathetic response. The HADITHI films would be played during the teacher-training to provide realistic scenarios for discussion. In social learning theory, parasocial interaction, where people begin to identify with and think of fictional characters as if they were real people, is a key concept in moving people towards behavioral change [24].

#### Case scenarios and teacher role play

The teacher role play and case scenarios are meant to help teachers better identify and navigate scenarios they may encounter in the classroom and schools in order to consider strategies for promoting positive and supportive environments for ALWH. Having teacher participants play the role of an individual living with HIV in these scenarios is meant to facilitate a connection to the lived HIV experience. In addition, modeling these interactions creates skills and then builds self-efficacy in teachers’ views of their own ability to navigate discussions with ALWH. The study team will read a common HIV-related scenario aloud to the teacher-participant group in order to facilitate a meaningful conversation about how best to proceed under those circumstances. In the role play portion of the training, teacher participants would be given a prompt and asked to act out the scenario using the skills they’ve learned in the training. During the exercise in front of the wider group, teacher participants play the role of the teacher and the peer navigator living with HIV plays the role of the ALWH. Following the role play, the presenters would ask the group to identify what was done correctly, what could be improved, and then open up a discussion about previous experiences and alternative approaches to the same scenario.

#### Experience sharing

The experience sharing portion of the teacher-training is meant to provide teachers real-life experiences of HIV stigma in the classroom directly from someone living with HIV. This portion, drawing on the increased intimacy created by self-disclosure, was meant to facilitate empathy from teacher participants and humanize the HIV experience. An adolescent peer-navigator living with HIV on the study team will speak to the teacher-participant group about their own experiences, facilitate conversation as appropriate and answer questions from the group.

#### Planned intervention

This teacher training module was being designed for evaluation in a cluster-randomized trial involving teachers from 20 schools ( 10 primary schools and 10 secondary schools) in Turbo and Ainabkoi-Sub Counties in western Kenya. Both the primary and secondary schools will be randomly selected from the sub-counties and randomized to either an intervention or control group. Teachers from the 10 randomly selected schools in Turbo Sub County will undergo training using the teacher training module developed above. The control group teachers are scheduled to have “usual”, Ministry of Health-supported teacher training sessions during this 6 month period and all schools had had a revised Kenya National AIDS and STI’s Control Programme (NASCOP) HIV educational curriculum incorporated into their teacher training and curriculum in the year prior to the intervention. All teachers at the same school will receive the same intervention assignment. The intervention group would have their HIV-related K/A/B evaluated immediately before the training, immediately after the training and then in a post-test at 6 months after the intervention. The control group teachers would have their HIV-related K/A/B evaluated at baseline and then in a post-test at 6 months. In addition, we planned to evaluate the experiences and clinical outcomes of ALWH and learning in the classrooms of the intervention and control group teachers through a separate, clinic-based assessment six months after the training intervention.

### Study design

Key informant interviews using cognitive interviewing techniques were used to assess how the teacher training module would be received, in terms of participants’ acceptance of concepts, ability to understand the terms, the perceived importance of the intervention, appropriate audience and suggested improvements [25]. We conducted key informant interviews with subject matter experts (SMEs) who included teachers, school administrators, healthcare workers, and adolescents. The SME were asked to view the films and evaluate the intervention materials described in Table 1, as well as to evaluate the planned procedures for holding the training, study recruitment and follow-up. The SME were asked to assess potential cultural and language barriers in the materials or approach, the acceptability among teachers as well as issues related to gender [25]. We utilized cognitive interviewing strategies to explore the validity, comprehensibility and relevance of the intervention [25]. The SME were identified through a snowball sampling technique and the adolescents were recruited within the AMPATH clinical system through purposeful sampling. We recruited an additional 14 adolescents for an adolescent community advisory board (ACAB) to review and provide feedback on the teacher training module.

### Setting

This study was conducted at the Academic Model Providing Access to Health care (AMPATH) program in Uasin Gishu County, located in Western Kenya. AMPATH is a long-standing partnership between a consortium of North American academic medical centers and Moi University in partnership with the Kenyan Ministry of Health that provides comprehensive care for over 160,000 people living with HIV across western Kenya, as well as comprehensive chronic disease management and primary care [26].

### Data collection and analysis

Interviews were conducted in Kiswahili or English, audio-recorded, translated and transcribed. Deductive thematic analysis was led by two investigators (AC and WB), involving line-by-line review of transcripts to identify meaning. These investigators independently extracted and compared results using the software program, Dedoose (SocioCultural Research Consultants, LLC). Along with a third investigator (JA), the research team reviewed transcripts several additional times to revise the coding structure as needed and compared and collapsed results based on consensus across the three analysts (AC, WB, JA).

### Ethical approvals

This study was approved by the Icahn School of Medicine at Mount Sinai Institutional Review Board, New York, NY, USA, and the Moi University / Moi Teaching and Referral Hospital’s Institutional Research and Ethics Committee in Eldoret, Kenya. Additional approval was received by the National Commission for Science, Technology, and Innovation (NACOSTI), a Kenyan government research regulatory body. Participants provided written informed consent before beginning interviews, and were given time to ask questions.

## Results

### Participant demographics

We recruited 50 SMEs (58% female, average age 34 years) including 15 primary and secondary teachers (60% female, average age 44 years), 6 school administrators (33% female, average age 51 years), 10 healthcare providers (70% female, average age 40.5 years), and 19 Kenyan adolescents between the ages of 12–21 years of age (9 ALWH and 10 HIV negative adolescents, 47% female, average age 17 years) to review the training package and provide feedback. Most of the SMEs reported knowing someone who was living with HIV. The ACAB was comprised of both HIV-infected and non-infected adolescents (42% female, average age 20.8 years).

### Feedback on module sections

Stakeholders provided feedback on the individual teacher-training module sections. (Table 2) Participants valued the idea of teacher discussions and role-plays, citing the potential for them to further grasp the concept among a larger group of their peers. Facilitating role-play scenarios in which teachers could learn and practice how to navigate challenging interactions with students was viewed as a valuable experience, one that would expand the teacher’s perspective. The SMEs agreed that the module sections were effective in communicating the intended messages, as the films depicting HIV stigma experienced by youth were a true reflection of their everyday life, the animations removed potential discrimination from real-life stories and the booklet identified common myths and misconceptions. The SMEs enjoyed the section on myths and misconceptions, all of whom expressed having heard these myths within their communities. The SMEs found all of the module sections to be culturally relevant and addressed the needs and stories of the average person in this setting, recognizing that each would also have unique experiences. The local context of the films, in particular, were seen as culturally relevant by the participants.

**Table 2:** Feedback on Module Sections

Sub-Theme	Illustrative Quote
*General support of the curriculum*	*‘According to me, the way you’ve come up with that solution like going to schools and making teachers to teach on stigma in schools, I think it will help a lot.’- HIV negative adolescent* *‘It really improves people’s knowledge about the disease because most of the things we hear about HIV are usually just the stigma and AIDS but not a lot people can actually tell you what is true with HIV and it’s a great learning opportunity for the people who haven’t heard the truth about HIV.’- HIV negative adolescent* *‘Yes, according to what I have gone through, I think I support the themes. Personally it actually touched me and how we always interact with the students and I think the training will be worth it.’- Secondary school teacher*
*Teacher group discussions/ Role play*	*‘I think that’s [role play] the best way to digest what they’ve seen. And in the process of sharing they get to get so many scenarios that exist out there. It might not have happened in their setting, but you never know maybe in the near future if it happens then they can be in a better position to handle it.’- Secondary school teacher* *‘You know one of the ways to diminish stigma is to talk about something and the fact that they will be doing it [teacher group discussions] among themselves … You are doing it among people who you don’t really know where they stand with the stigma, so it’s a way of you to get comfortable about talking about it and that in a way to me is a way of reducing stigma.’- Social worker*
*HIV Booklet: Myths and misconceptions*	*‘I really liked the one about the myths because we still do get some kids who talk about like mode of transmission being deep-kissing and that has to come from somewhere, it has to come from school.’- Mental health counselor* *‘I have always had a negative attitude towards HIV but once I get this message, what is explained here can really change my thinking-- “ooh that HIV is not a killer disease after all”.’- Clinician* *‘Yeah the booklets contain the information about all the facts and the myths about HIV. So when the people are equipped with the facts, I believe it will reduce the stigma.’- Education administrator* *‘I liked the part on relationships. Because that is where it’s being talked about. It is really giving information to the adolescents where they are having a lot dilemmas.’- Social worker*
*HIV films*	*‘What I liked most about the videos is that they are very brief, and they are passing their message so clearly. There is no time that is wasted there. They are on point and precise.’- Social worker* *‘The videos make it effective because they really show the real life of these children in school, at home and the other places where they interact in the field.’- Primary school teacher* *‘These videos are actually a true reflection of what is happening in our society, what happens when we realize people have HIV/AIDS -- not everybody loves you, and some will love you. So I think it is actually from a natural environment, what takes place in our environment. So that training will serve us at its best because we live in that environment.’- Secondary school teacher*
*HIV animation*	*‘The animation, I liked it because the visual impression is very important because even in teaching, the visual impression helps [more] than things of just sound. So that when they hear the voice and see, the understanding goes deeper.’- Secondary school teacher* *‘Animations are good because you don’t need to use people’s images. So it is good and it is non-discriminatory. There is no stigma with animation.’- Clinician*
*Cultural relevance*	*‘The setup is just within our own vicinity. So it shows that these things are not happening elsewhere they are just happening within our own society and our own community.’- Clinician* *‘As much as they were acting, they sounded real. I mean these are things that happen. If you wouldn’t have said that they are acting, I would have gone home believing it is real life. That is, if you would not have said anything. They pass the message till you can actually feel them.’- Adolescent community advisor board* *‘Yes. Actually that scenario was a very realistic one. I have even experienced that. I have seen where someone who knows about the status of a teenager goes round telling others and that causes stigma. So teachers can relate that by really understanding the learner, the background of the learner and trying to assist where appropriate.’- Secondary school teacher*
*Ability to Understand*	*‘The messaging, the language, the vocabulary itself, I think it’s self-explanatory.’- Clinician* ‘*Like I’ve mentioned before, simplicity. It’s down to earth, it can handle those in the lower strata of the society and those in the upper strata. Then, simple language is used.’- Secondary school teacher*

### Experiences with stigma and perceived impact of module

SMEs also described significant and diverse experiences of HIV stigma in this setting (Table 3), and believed that adolescents also experienced significant HIV stigma from their primary caregivers. The participants identified several areas in which the teacher training module may make a critical impact on HIV stigma, and supported the planned intervention, inclusive of the underlying theory. The SMEs reported that the training module was resourceful in providing information about HIV and its mode of transmission and treatment, all of which may shape teacher knowledge through the facilitation of accurate HIV education. SMEs acknowledged that incorrect knowledge may negatively influence the methods by which teachers educate their students, contributing to HIV stigma in this setting. The potential for the training module to increase awareness of the prevalence of HIV stigma overall was identified, as well as the importance of teachers and caregivers in providing support and guidance of ALWH. Lastly, participants thought that providing HIV education in this format may sensitize the audience to topics related to HIV, therefore normalizing the experiences of those affected.

**Table 3:** Experiences with stigma and perceived impact of module

***Theme***	***Illustrative Quotes***
*Experiences with stigma*	*‘It is because some or even the adults and some other teachers whom I’ve mixed with, when they hear HIV they get scared.’- Secondary school teacher* *‘So because I know even some of the people even in the church and where I’ve mixed, when they hear of HIV they leave.’- Secondary school teacher* *‘There’s a family we visited and the grandmother could just shout at the children talking the same way the video was talking about, “You will infect us”, “This your drugs!” You know when you talk about your drugs, you are already stigmatizing the child, so the videos are real life situations and that is really what is happening on the ground.’- Social worker* *‘Okay, even there is a time, time for inspections, in boarding schools, I also had that problem. I could lock my box and tell my cube mates, tell them that I’m not around I’ve gone to …I’m sick I’ve gone home, but I’m around. Teachers don’t understand why you carry those drugs. So they remove it, they expose it, they ask you what they are for. They once caught me with those drugs, I had to fake a disease like I said I have chest problems and headaches, so this is the drugs I’m using. But it was, okay I didn’t have any other way to do it.’- HIV negative adolescent*
*Caregiver Role*	*‘In home environment, teach the parents to love their children, to create a positive attitude in children, to support them in all ways.’- Primary school teacher* *‘Because I know by the end of the day when the other parents get to hear about this, they wouldn’t want their child to play with this [infected] child, so they can reach out to all these other children and try to give them what is factual and their role, they need to play a role in supporting this child.’- Social worker* *‘Again with disclosure, I feel also that the caregivers should be told and be made aware that this student needs to be told. As a teacher I am talking from experience whereby there are some pupils now they are in grade eight, they don’t know why they are taking their medication.’- Primary school teacher*
*Teacher role*	*‘You can use the teachers, then the teachers will empower the parents, then the parents will take care of their children.’- Adolescent living with HIV* ‘T*hey’ll help the student suffering from HIV to be able to conduct their day to day routines and also be able to take their prescriptions every time without concealing the secret to the students or to any other staff in the school.’- Social worker* *‘So teachers are the ones who should instill in the youth that anybody who is positive, there is nothing wrong, he/she is normal and he does everything and cannot just pass that condition like that.’- Secondary school teacher*
*Perceived Impact of the module on stigma*	*‘I think they will understand HIV better as a situation not as a disease that can kill. Then I think they will be able to help any student who is dealing with stigma and all that. They can help them to deal with it positively and change the attitudes towards themselves and how they relate with other people.’- Secondary school teacher* *‘So I believe teachers when we watch such video and get information about what I have watched, we’ll be able to change the way we teach and the way we handle students.’- Secondary school teacher* *‘If the teachers and the children can all be reached, the perception about HIV will definitely change.’- Primary school teacher*
*Lessons learnt*	*‘I have learnt that some of the teachers, parents and some members of the society, we are the people who cause this stigma to these children’- Primary school teacher* *‘It made me aware of the barriers that our adolescents face, sometimes we take it too lightly or we think the problem lies with the adolescents, but you could have somebody who is so willing and so committed to their care and treatment and yet certain things at home are really holding them back.’- Mental health counselor* *‘That a person living with HIV is not a wicked person in the community and infection of HIV virus is not the end of life. That a person may be able to live many years with HIV as long as the medication is concerned and also the clinics are available.’- HIV negative adolescent*

### Suggestions for improvement

The participants provided important feedback for improving the teacher-training module. (Table 4) The module was presented to the SMEs in English, after which most participants suggested creating another version in Kiswahili for teachers or community members with limited English proficiency. The majority of the participants recommended visual changes to sections of the training module, including adding more color and pictures to the paper resources. Although the videos included in the training-module were perceived as culturally relevant and a good representation of reality, almost half of participants suggested including real-life situations, either through additional videos with PLWH or including an ALWH in person to share their experiences with the group. SME’s also suggested highlighting famous or well-known PLWH in the training to demonstrate that PLWH are able to live long and healthy lives. Lastly, much of the teacher-training module sections incorporated the experiences of male children and ALWH.

**Table 4:** Suggestions for Improvement

*Language considerations*	*‘We can maybe have a Kiswahili translation of it for those who may not be understanding the English language comfortably or even the vernaculars.’- Secondary school teacher*
*Visual changes*	*‘The animation. It was good I think you should just add some a bit of color for it to be appealing to the eyes.’- HIV negative adolescent* *‘I was imagining it should be more of pictorials added because especially us Kenyans, reading a whole story that is written in this manner, most people do not have that patience. A pictorial could have serviced.’- Secondary school teacher*
*Using real situations*	*‘Also the time that you will be presenting, how about you bring someone who is HIV positive to share his/her story with them?’- Adolescent community advisory board* *‘Now what we can only add here is, maybe you can give examples of people who have lived with the disease for a certain number of years. They have lived healthy for certain number of years, they have been living healthy throughout their lives, they have lived with the disease, and they have lived up to a hundred years or something like that.’- Primary school teacher* *‘I think also the best way we can do it, if we have people or adolescents, they share the real life experience, so that the other people can know it is, this thing is real. The experience. They share what they normally go through, I think it can also help teachers to understand that this thing is real how they are being discriminated in school, how they want to be handled in school, I think when we have people with the disease they explain their life experience, it can really help.’- Nurse*
*Gender differences*	*‘My worry initially was that the first two examples is for boys. I was getting worried, what about teachers who handle girls in a school setting and what have you.’- Secondary school teacher* *‘We can still add another one like a girl, so that the girls can still express...you know the challenges that the boys are passing, and the challenges the girls, you know girls have more challenges than boys. If we can still add the video for the girls.’-Nurse*

Some participants expressed the need to include more narratives and experiences of female ALWH. The SMEs articulated the need for teachers to better understand how to navigate scenarios with female students living with HIV, as they perceived girls to experience more HIV-related challenges. One participant feared that girls may come to believe that only boys can become infected with HIV if only boys were showed in the training. Additionally, SMEs requested having both female and male ALWH present during the teacher-training, so that both perspectives were present and so that there was opportunity to address gender-specific needs.

### Expansion of the training

All SMEs articulated the importance of expanding the proposed teacher-training module outside of the originally intended population and setting. A wide range of populations were identified as important audiences for the training, including parents of adolescents, government and religious officials, community leaders and law enforcement agencies. Reducing HIV stigma was perceived as a community effort for which all people should be involved.

*‘I believe it is important for the society as a whole because we all relate in one way or another. From the shopkeeper to the guardian to the teacher, to the support staff in the school, everyone is really responsible for the stigma.’- Secondary school teacher*

*‘But also people in the community I think. Even in the church and all that because you'll find that some people use their religion as a source of comfort, and you know a place where they are free of judgment and all of that.’- Mental health counselor*

*‘Yeah, because the teachers are with the students from 8-5. For the cases that of schools that are day schools, the parents and the guardians will be with them from 5 until the following day and during the holidays. So this information will be very crucial for the parents too.’- Secondary school teacher*

Participants suggested adapting the teacher-training module for use on social media platforms like Facebook and WhatsApp, and for viewing on the television and in newspapers. For individuals in rural communities who may have limited access to these approaches, in-person community meetings were suggested for presentation of the module.

*‘You can sell them to the news people in the television or even you can put them in the newspapers for people to read.’- Adolescent living with HIV*

*‘For example the county government can call for a public meeting and then do the awareness [training].’- HIV negative adolescent*

*‘I believe that animation can be used most effectively by sharing it. You know nowadays people embrace technology and most people are on the social media. It can be shared on WhatsApp, Facebook.’- Secondary school teacher*

## Discussion

We present a description of the rigorous development and cultural adaptation of a teacher training module that could be used with primary and secondary school teachers in East Africa with the intent to reduce teachers’ stigmatizing K/A/B related to HIV and increase positive A/B towards HIV. We hypothesize that implementation of the training will facilitate a critical empathy response among participants, which will subsequently remove barriers to connection with PLWH. We anticipate that the downstream effect of more meaningful and accurate connections to PLWH is less stigmatizing attitudes and behavior toward this population. This training course incorporated elements from a critical literature and curriculum content review, as well as integration of our team’s prior, novel work to develop HIV-related educational and counseling materials and multimedia that were created collaboratively with community partners in Kenya. The module incorporates components like role play scenarios, case scenario discussions, educational videos and lectures, as described previously in the literature, and will also include real-life perspectives from peer educators living with HIV, a component encouraged and supported by the SMEs who evaluated the module within this study. We also present the findings of interviews with education sector stakeholders to provide critical feedback on the proposed teacher training module. Our data demonstrate significant acceptability of the teacher training module among education sector SMEs and adolescents, both HIV positive and negative.

All SME participants stressed the importance and need for stigma reducing interventions in the classroom, highlighting their own experiences observing stigmatizing behaviors in the community. The participants perceived the training as culturally relevant and easy to understand and had minor suggestions for improvement, including using image-based resources and brighter colors for ease of reading. The importance of including the myths and misconceptions section of the teacher-training module was discussed, citing that it provided accurate HIV related information and dispelled false perspectives of HIV infection. This data is consistent with studies demonstrating gaps in HIV knowledge and subsequent negative attitudes toward ALWH, leading to increased experiences of stigma for individuals living with HIV and acceleration of stigmatizing behavior regarding HIV programs and education in the school setting [27, 28]. Teachers’ perception of HIV has been identified as a key factor in the adoption of HIV programs and education in schools, as their knowledge and beliefs may affect the content and quality of their teaching, directly affecting stigma prevalence in the classroom [29].

Little is known about HIV perception and stigmatizing behaviors of teachers in Kenya, as many rely on Ministry of Education approved syllabi to disseminate HIV information in the classroom. The context in which this information is relayed in the classroom is dependent entirely on the attitude of the teacher [15]. The SMEs believed that engaging teachers in role-play scenarios would be a beneficial approach in normalizing HIV-related experiences and providing critical assistance and support in navigating challenging situations with their students. We note as a limitation that this study evaluated stakeholder perspectives on the planned intervention, and therefore did not assess the impact of said intervention on the target study population. Additional studies are needed to investigate whether targeted evaluations such as this teacher training module can have a long-term impact on the HIV knowledge, attitudes and beliefs manifested by teachers in their classroom.

## Conclusion

There is a significant gap in understanding the ability to enrich teacher K/A/B through interventions to reduce HIV stigma in the classroom. Key stakeholders confirmed the importance and significant need to reduce HIV stigma in this setting. Data from interviews with education sector stakeholders demonstrate that our process for developing a culturally appropriate multi-media intervention to reduce HIV stigma in the classroom was feasible.

## Supplementary Information

**Additional file 1.** Teach HADITHI SME Interview Guide. The study team created this interview guide to be used with subject matter expert participants enrolled in this study.

## Data Availability

The datasets used and/or analyzed during the current study are available from the corresponding author on reasonable request.

## Abbreviations

ALWH: Adolescents living with HIV
K/A/B: Knowledge, attitudes and beliefs
SME: Subject matter experts
HIV stigma: HIV and AIDS related stigma
FGD: Focus group discussion
AMPATH: The Academic Model Providing Access to Healthcare
