# Supplementary material for: Perspectives of education sector stakeholders on a teacher training module to reduce HIV/AIDS stigma in Western Kenya
Source: BMC Public Health. 2021 Jun 30;21:1281. doi: 10.1186/s12889-021-11331-5 (PMC8247165; doi:10.1186/s12889-021-11331-5)
Supplement: Supplementary file 1 — Additional file 1. Teach HADITHI SME Interview Guide. The study team created this interview guide to be used with subject matter expert participants enrolled in this study. [file 12889_2021_11331_MOESM1_ESM.doc]

Semi-structured interview guide after participant has review teacher training curriculum materials:

1. Demographics

- What is your age?
- What is your occupation?
- If you are comfortable, would you share your HIV status with us?
- Do you have family members or friends who are living with HIV?

2. HADITHI films

- What was your first impression about the HADITHI films?
- What were the major messages that you took away from the HADITHI films? In other words, what do you think the films were trying to say?
- Do you think these films were effective in getting this message across? Why or why not?
- Do you think these films could be used to improve people’s understanding and knowledge of what it is like for a child to be living with HIV today?
- Do you think these films could be used to reduce stigmatizing beliefs about HIV?
- What did you like most about these films?
- What did you like least about these films?
- Who do you think these films would be most appropriate to share with?
- Did the terms and the way things were explained make sense to you? Or could it be improved?

3. HIV animation

- What was your first impression of the HIV animation?
- What were the major messages that you took away from the HIV animation? In other words, what do you think the animation was trying to say?
- Do you think the animation was effective in getting this message across? Why or why not?
- Do you think this animation could be used to improve people’s understanding and knowledge of how HIV works, how HIV treatment works, and how HIV is spread?
- Do you think the animation could be used to reduce stigmatizing or incorrect beliefs about HIV, its treatment, and the way it is spread?
- What did you like most about the animation?
- What did you like least about the animation?
- Who do you think the animations is most appropriate to share with?
- Did the terms and the way things were explained make sense to you? Or could it be improved?

4. HIV FAQs booklets

- What was your first impression of the HIV FAQs booklets?
- What were the major messages that you took away from the HIV FAQs booklets? In other words, what do you think the booklets were trying to teach you?
- Do you think the booklets were effective in getting this message across? Why or why not?
- Do you think this booklet could be used to improve people’s understanding and knowledge of how HIV works, how HIV treatment works, and how HIV is spread?
- Do you think the booklets could be used to reduce stigmatizing or incorrect beliefs about HIV, its treatment, and the way it is spread?
- What did you like most about the booklets?
- What did you like least about the booklets?
- Were there additional questions that you or other people might have about HIV that should be included in these booklets?
- Who do you think the booklets are most appropriate to share with?
- Did the terms and the way things were explained make sense to you? Or could it be improved?
